# Horizontal Inequity in Health Care Utilization among the Middle-Aged and Elderly in China

**DOI:** 10.3390/ijerph14080842

**Published:** 2017-07-26

**Authors:** Chaofan Li, Lei Dou, Haipeng Wang, Shanshan Jing, Aitian Yin

**Affiliations:** 1 School of Health Care Management, Key Laboratory of Health Economics and Policy Research, NHFPC, Shandong University, No. 44 Wenhua Road, Jinan 250012, China; lcfan1129@163.com (C.L.); doulei@sdu.edu.cn (L.D.); wanghaipeng@sdu.edu.cn (H.W.); 2 School of Traditional Chinese Medicine, Shandong University of Traditional Chinese Medicine, Jinan 250355, China; michelle.84@live.cn

**Keywords:** horizontal inequity, health care utilization, middle-aged and elderly, China

## Abstract

Background: Equitable utilization of health care is a primary goal of the Chinese health system. This study aimed to examine horizontal inequity in health care utilization and identify the factors resulting in inequity among the middle-aged and elderly in China. Methods: The data were obtained from the China Health and Retirement Longitudinal Study (CHARLS). We employed the concentration index (CI) and horizontal inequity (HI) to measure inequity in health care utilization. Non-linear regression models were used to decompose the CI into the contribution of each factor. Results: The CIs for the probability of and total number of outpatient visits were 0.0642 and 0.0780, respectively, and those for inpatient visits were 0.1418 and 0.1471, respectively. The HIs were also significantly positive. Living standard was the most important contributor. The contribution of health insurance varied between plans. Conclusions: This study supported the presence of pro-rich inequity in health care utilization for both probability and frequency among the middle-aged and elderly in China. Narrowing the living standard gap and improving the health insurance benefit package for the elderly should help to resolve these inequities.

## 1. Introduction

Equity in access to and utilization of health care is a primary goal of health systems [1,2]. The principle of horizontal equity, which is defined as those in equal need ought to be treated equally, irrespective of their economic status, geographic location, or race, is a yardstick for international comparison [3,4,5]. Inequity in health care utilization has been reported in many countries [6,7,8,9,10,11,12,13], including China [2,14,15,16,17,18].

Equity in health care utilization for older people has become an important issue in the context of population ageing. Population ageing is likely to result in increasing health needs, particularly in low- and middle-income countries [19]. Various health problems, including hypertension and diabetes, are more common in older people than younger [20]. However, there are barriers for older people, such as unaffordability and lack of transportation, to getting access to health care. In light of the challenges of population ageing, eliminating barriers to health care utilization and improving equity is important. Population ageing in China, characterized by “growing old before growing rich” and by a rapidly ageing population, poses a considerable challenge. In 2012, there were 194 million older people aged 60 and above, constituting 14.3% of the total population in China. The absolute size and percentage of the elderly population is predicted to be 483 million and 34.1% of the total population by 2050 [21]. Improving health equity in China is increasingly important, especially in the context of population ageing.

To address the widely acknowledged inequity in health and health care utilization, the Chinese government initiated a new round of health care system reforms in early 2009. Policy measures, including the expansion of health care coverage and reduction of out-of-pocket payments, were introduced. Three health insurance plans, the Urban Employee Basic Medical Insurance (UEBMI), New Rural Cooperative Medical Scheme (NRCMS), and Urban Residents Basic Medical Insurance (URBMI), have been established to improve equal access to health care. The majority of older adults had enrolled in at least one health insurance plan by the end of 2011. Although most older people were covered, obvious coverage gaps existed between the different health insurance plans. All of them covered both outpatient and inpatient health care and the URBMI and NRCMS had low ceilings for outpatient visits. Additionally, the reimbursement rates for inpatient visits offered by UEBMI, URBMI, and NRCMS were 69%, 54%, and 50%, respectively, in 2013. Overall, the UEBMI provided more health benefits to enrollees than URBMI and NRCMS.

Equity in health care utilization among the elderly has been studied in some countries. As Cebada and Garrido showed, there were pro-poor inequalities in both the probability and conditional number of general practitioner (GP) visits in Spain [22]. Conversely, Joe and his colleagues found that horizontal inequity in health care utilization among the elderly was pro-rich in India [23]. Terraneo also identified substantial educational inequality in utilization of health care in Europe [24]. Both Wang and Zhang found that pro-rich inequity existed in the probability of health care utilization among the middle-aged and elderly in China [25,26].

The decision process in physician utilization consists of two stages. To some extent, patients decide whether to seek for a physician, but once a physician has been contacted, the physician determines repeat visits and referrals [5]. The subsequent number of health care visits is more likely to be influenced by physicians than by patients. The distinction between the probability and the frequency of health services is important as it generates further insights into the ways that utilization patterns differ. Total inequity can be broken into inequity in the probability of a visit and inequity in the conditional number of visits. Measuring the horizontal inequity in health care utilization for both probability and frequency can highlight whether an observed pattern of inequity is patient-initiated or doctor-driven. Because few studies of health utilization inequities in older people have been done, distinguishing whether a greater proportion of the observed horizontal inequity originates from patient-initiated or doctor-driven care utilization is important [2].

The purpose of this study was to examine the inequality and horizontal inequity in health care utilization by the middle-aged and elderly in China, focusing on both probability and frequency. We also attempted to identify the factors contributing to the observed inequality and inequity.

## 2. Materials and Methods

The data were obtained from a publicly available database [27], the China Health and Retirement Longitudinal Study (CHARLS), which was conducted by the Institute of Social Science Survey, Peking University, from July to August 2013. Follow-up data are collected every two years, to obtain a high-quality, nationally representative sample of Chinese residents aged 45 years and older that will meet the needs of scientific research on the elderly. It includes a wide range of topics including demographics, health status and functioning, health care and insurance, work, income and consumption, assets (individual and household), community level information, etc. Using a four-stage probability sampling method, 18,605 individuals aged 45 years and over were sampled from 10,803 households in 28 provinces. In the first stage, 150 county-level units were randomly chosen with a probability-proportional-to-size (PPS) sampling technique from a sampling frame containing all county-level units with the exception of Tibet and stratified by region and then by urban districts or rural counties and by per capita gross domestic product. In the second stage, three primary sampling units (PSU) consisting of administrative villages (cun) in rural areas and neighborhoods (shequ or juweihui) in urban areas, were selected within each county-level unit, using PPS sampling. Households living in the eligible dwellings were included, except for empty or non-resident dwellings. Last, the eligible individuals aged 45 and older were interviewed. When the data in CHARLS was compared with the data in the Chinese population census of 2010, the CHARLS sample was quite similar to the Chinese national population [28]. Face-to-face computer-assisted personal interviews were conducted by well-trained interviewers. This study used a cross-sectional design by utilizing the CHARLS data for 2013. The response rate was 71.5% and a total of 13,302 individuals were eventually included.

Health care utilization was measured by four variables: (1) The probability of outpatient visits in the last month was measured by the question “In the last month have you visited a public hospital, private hospital, public health center, clinic, or health worker’s or doctor’s practice, or been visited by a health worker or doctor for outpatient care?”; (2) The total number of outpatient visits in the last month was measured by the question “How many times did you visit/been visited in the last month?”; (3) The probability of inpatient visits in the last year was measured by the question “Have you received inpatient care in the last year?”; (4) The total number of inpatient visits in the last year was measured by the question “How many times have you received inpatient care in the last year?”.

To decompose total inequality with a consistent method, the independent variables were classified into three groups: (1) need variables, (2) living standard, and (3) non-need variables. Need is a rather elusive concept that has been interpreted in relationship to the definition of equity in health care delivery [29]. We defined health care need in terms of the patient’s health and disease status, as measured by their self-assessed health status, chronic disease, and disability. We also adopted gender and age as proxy need measures, since health care need is often gender- and age-specific. The measurement of self-assessed health status was based on the question: “Would you say your health is very good, good, fair, poor, or very poor?”. Age was divided into three groups: 45–59, 60–74, and 75+ years. Fourteen kinds of chronic diseases were identified: hypertension, dyslipidemia, diabetes or hyperglycemia, cancer or malignant tumor, chronic lung disease, liver disease, heart disease, stroke, kidney disease, stomach and other digestive disease, emotional nervousness or psychiatric problems, memory-related disease, arthritis or rheumatism, and asthma.

Per capita household expenditure (pce) was used as a proxy for living standard, as income calculated from the CHARLS data may contain errors and thus would not imply commensurate changes in living standard. Moreover, consumption expenditure is a better proxy as it is easy to measure and closely linked with well-being [17]. We used the natural logarithm value of the per capita household expenditure (ln pce) to measure living standard and grouped individuals into five groups, from poorest to richest.

Six non-need variables were considered. First, UEBMI, URBMI, and NRCMS were the primary health insurance plans in China. Second, the 28 provinces were divided into three regions, eastern, central, and western China, according to the China Statistical Yearbook of Health and Family Planning. Third, we divided the occupations into four groups, including agricultural work, employed, self-employed, and not working. Finally, the other non-need variables included education (illiterate, primary school, middle school, high school, and college and above), marital status (married, divorced/widowed, and unmarried), and living residency location (rural and urban).

We employed a concentration index (CI) to measure socioeconomic-related inequality in health care utilization. The CI quantifies the degree of socioeconomic-related inequality in health variables. It is sensitive to the population distribution across socioeconomic groups and considers the socioeconomic dimension in health care inequality [30]. Thus, it has been used as a standard tool to measure and compare the degree of inequality in health care utilization. Concentration curves plot the cumulative proportion of the population (ranked by living standards, beginning with the lowest group) against the cumulative proportion of health variables [31]. Following Wagstaff, the CI is defined as twice the area between the concentration curve and the diagonal (the line depicting equality). The CI ranges from −1 to 1. When the concentration curve lies above (below) the diagonal, the CI shows a negative (positive) value, indicating inequalities in health favoring the poorer (richer) members. In other words, if the CI is negative (positive), it indicates that the disproportionate distribution of health care utilization is more concentrated among the poor (rich) and is called “pro-poor” (“pro-rich”) inequality. No inequality exists in the health variables only if the concentration curve coincides with the diagonal. The CI can be written as

(1) $$\mathrm{CI}=\frac{2}{N\mu }\overset{N}{\underset{i=1}{\sum }}h_{i}R_{i}-1-\frac{1}{N}$$

where $h_{i}$ is the health sector variable, μ is its mean, and $R_{i}$ = iN is the fractional rank of an individual *i* in the distribution of living standards (*i* = 1 for the poorest and i = N for the richest). Generally, for computational convenience, another formula for the CI defines it in terms of the covariance between the health variable and the fractional rank in the living standards:

(2) $$\mathrm{CI}=\frac{2}{\mu }\mathrm{cov}\left(h,R\right)$$

Horizontal equity is defined as equal treatment for equal need, irrespective of other characteristics, such as socioeconomic status or place of residence. There is horizontal inequity when individuals with same need have a different amount of health care. CI measures the extent of socioeconomic-related inequality in health care utilization. However, because of differences in the need for health care between different socioeconomic groups, inequality does not reflect inequity. To measure inequity in health care utilization, we applied the horizontal inequity index (HI) [31]. HI estimates the CI for need-standardized health care utilization. The need could be represented by gender, age, self-assessed health status, and chronic diseases (measuring and testing). Wagstaff and van Doorslaer proposed to measure HI by the gap between the inequality in the actual and needed use of health care:

(3) $$\mathrm{HI}=\mathrm{CI}-{\mathrm{CI}}_{N}$$

where ${\mathrm{CI}}_{N}$ denotes the CI corresponding to the need-predicted utilization of health care. Similar to CI, a negative (positive) value for HI indicates horizontal inequity favoring the worse-off (better-off).

The decomposition method proposed by Wagstaff was used to explain the socioeconomic-related inequality in health care utilization and to measure HI [32]. This is a straightforward method used to decompose inequality into the contributions of various explanatory factors. For nonlinear models, decomposition is possible only if some approximation is made. The approximation error of a nonlinear model tends to be smaller and a nonlinear decomposition represents a somewhat closer approximation of the partial contributions than a linear decomposition does. In the nonlinear approximation explanatory model,

(4) $$h_{i}=G\left(\alpha^{m}+\delta^{m}y_{i}+\underset{j}{\sum }\beta_{j}^{m}x_{ji}+\underset{k}{\sum }\gamma_{k}^{m}z_{ki}\right)+\varepsilon_{i}$$

where $\alpha^{m}$ is the intercept, $\delta^{m}$, $\beta_{j}^{m},$ and $\gamma_{k}^{m}$ are partial effects, y represents the living standard, $x_{j}$ represents the need variables (gender, age, self-assessed health status, chronic disease, and disability), and $z_{k}$ denotes the non-need variables (education, occupation, residency, region, and health insurance); $\varepsilon_{i}$ is a residual term; and $h_{i}$ is the health care utilization variable. In this equation, y, $x_{j}$, and $z_{k}$ are determinants for health care utilization; G will take particular forms for the probit or general negative binomial model. Given the relationship between h and y, $x_{j}$, and $z_{k}$, CI can be written as

(5) $$\mathrm{CI}=\left(\frac{\delta^{m}\bar{y}}{\mu }\right){\mathrm{CI}}_{y}+\sum_{j}\left(\frac{\beta_{j}^{m}\bar{x_{j}}}{\mu }\right){\mathrm{CI}}_{j}+\sum_{k}\left(\frac{\gamma_{k}^{m}\bar{z_{k}}}{\mu }\right){\mathrm{CI}}_{k}+\frac{{\mathrm{GCI}}_{\varepsilon }}{\mu }$$

where μ is the mean of the health care utilization variables, $\bar{y}$ is the mean of the living standards, ${\mathrm{CI}}_{y}$ is the CI for the living standards, $\bar{x_{j}}$ is the mean of the need variables, ${\mathrm{CI}}_{j}$ is the CI for the need variables, $\bar{z_{k}}$ is the mean of the non-need variables, ${\mathrm{CI}}_{k}$ is the CI for the non-need variables, and ${\mathrm{GCI}}_{\varepsilon }$ is the generalized CI for $\varepsilon_{i}$.

We decomposed the CI for the probability of healthcare visits by the probit model and the total number of health care visits by the general negative binomial model. Each household was a cluster and the White-Huber-Sandwich estimator was used to correct for cluster sampling. Each CI was decomposed into partial contributions of the need contributors (gender and age dummies, self-reported health status dummies, chronic disease dummies, and disability dummies), non-need contributors (health insurance, education, married status, regions, occupation status, and area), and living standards. All the statistical analysis was conducted using STATA version 14.0 (StataCorp LP., College Station, TX, USA).

## 3. Results

### 3.1. Descriptive Results

Table 1 presents the characteristics of the study participants. Approximately one fifth (21.24%) of the population aged 45 and older had at least one outpatient visit in the last month, and the mean number of outpatient visits was 0.49. Of the respondents, 12.43% had utilized inpatient health services at least once in the last year, and the mean number of inpatient visits was 0.18. Half of the respondents reported that they had poor or very poor health status, with more than 70% of the sampled individuals suffering from chronic diseases and 22.93% reporting disabilities. Most of the middle-aged and elderly were enrolled in the NRCMS (68.41%), while about 3% of the respondents were not covered by any health insurance plan. The majority of the participants had a low education (64.60%) and lived in rural areas (61.36%). Likewise, the majority of the subjects were engaged in agricultural work (45.89%) or were not working (31.42%). Finally, the mean of the living standards was 11,177.52 Chinese Yuan (CNY).

### 3.2. Indices for Total Inequality and Horizontal Inequity

Table 2 shows the probability and total number of health care utilizations across the living standard quintiles and indices for inequality and horizontal inequity. The result shows that 26.50% of the richest utilized outpatient care during the previous month, while only 18.19% of the poorest did so. In addition, the number of outpatient visits ranged from 0.4055 in the poorest to 0.6372 in the richest. Moreover, the probability of inpatient care utilization in the richest (0.1725) during the previous year was almost twice that of the poorest (0.0888). The number of inpatient visits in the richest (0.2483) was also twice that of the poorest (0.1261). It shows an obvious inequality in the distribution of health care utilization across the socioeconomic groups.

The CIs for the probability (CI = 0.0642) and the total number of outpatient visits (CI = 0.0780), were both positive, which means that the better-off had more outpatient visits than the worse-off. The higher income groups were not only more likely to have had outpatient visits but were also more likely to be treated more frequently. Furthermore, the inequality indices for the total number were a little higher than the indices for probability. After controlling for unequal need distributions, we obtained the horizontal inequity (HI). The HIs for probability (0.0714) and total number (0.0882) were also significant, and there was evidence of pro-rich inequity in outpatient visits.

Similar to the inequality of outpatient care utilization, there was a pro-rich distribution of inpatient care visits. The CIs for probability and total number of inpatient visits were 0.1418 and 0.1471, respectively. After adjusting for need differences, the HIs for probability (0.1636) and total number of inpatient visits (0.1596) were also significantly positive. Moreover, the values of the indices for inpatient visits were much higher than those for outpatient visits.

### 3.3. Decomposition of Inequality in Outpatient Visits 

Table 3 shows the decomposition result for the inequality in the utilization of outpatient visits. The positive (negative) partial contribution indicates that the determinant increases (decreases) the total inequality in health care utilization, with positive (negative) percentages referring to increases (decreases) in percentages. This decomposition indicated that the dummy variable representing very poor health contributed to most of the pro-poor inequality (25.71%), while the presence of chronic diseases made a pro-rich contribution (5.16%). Living standard contributed to the majority of the pro-rich inequality (91.39%). The contribution of health insurance varied from plan to plan. Specifically, the NRCMS made a positive contribution (19.71%), but the URBMI made a negative contribution (−4.29%). Other need or non-need factors also had positive or negative contributions, as shown in Table 3.

With respect to total number of health care visits, most of the need dummy variables, except those for the elderly over 75, fair and good health status, and the presence of chronic disease showed negative contributions, ranging from 0.82% to 17.50%. Among the non-need variables, the URBMI and the other health insurance plans also showed negative contributions. However, this seemed to be offset by the pro-rich contribution of the NRCMS (8.26%). Again, living standard provided a positive and higher contribution to inequality, reaching 72.04%.

### 3.4. Decomposition of Inequality in Inpatient Visits

Table 4 summarizes the decomposition result for inequality in inpatient visits. The illness-related indicator contributed markedly to pro-poor inequality, whereas the presence of chronic disease had pro-rich contribution (1.70%). Among the non-need variables, not working and living standard contributed to a high pro-rich inequality. Their contributions were 10.69% and 93.45%, respectively. Moreover, the URBMI reduced the pro-rich inequality (−1.18%), whereas the NRCMS made a pro-rich contribution and expanded the inequality in probability of inpatient visits (0.31%).

For inpatient visits, the contributions of most need dummy variables, such as male elderly, poor health status, and disability, were negative, but the presence of chronic disease contributed to pro-rich inequality (2.07%). Non-need variables usually showed low negative contributions, while the occupation status of not working had a pro-rich contribution as high as 9.26%. The URBMI and NRCMS contributed −0.80% and 1.48%, respectively. The living standard showed a positive contribution as high as 77.19%.

**Figure 1:**
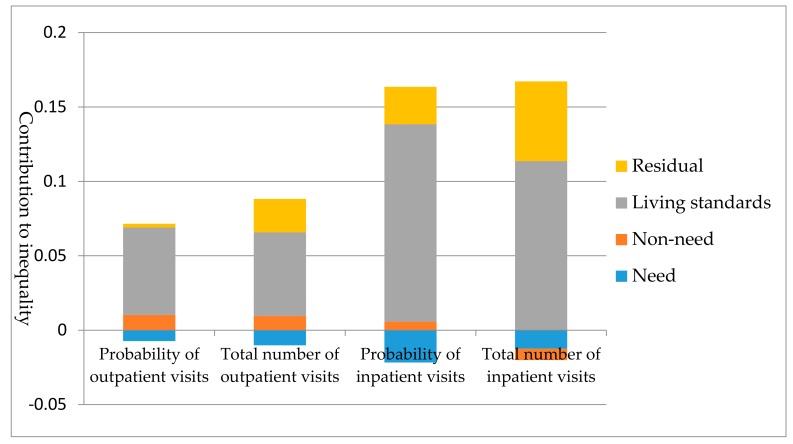
Decomposition of inequalities in health care utilization.

Figure 1 shows the contribution of inequality decomposed into the four main sources: need variables, living standard, non-need variables, and residual terms. The total contribution of all the need variables is the sum of the contributions of gender, age, self-reported health status, presence of chronic disease, and disability. The corresponding values for the four types of health service utilization were negative, −11.37%, −13.08%, −15.30%, and −8.43%, respectively. The contribution of all the need variables reduced the inequality. However, the distribution of pro-poor utilization driven by need factors was counterbalanced by the non-need contributors and the living standard. The living standard was the most important contributor, with a high positive contribution (ranging from 72.04% to 93.45%).

## 4. Discussion

This study provides new evidence about inequity in health care utilization among the middle-aged and elderly after the new round of health system reform in China. We found that a pro-rich inequality and horizontal inequity in probability of health care utilization existed. We also examined the inequity in frequency of health care utilization and found that the rich were not only more likely to utilize health care, but also had more frequent visits. The pro-rich inequity in health care utilization under the context of universal health insurance coverage violated the rule of “equal treatment for equal need”. Additionally, this study shed light on the relative contributions of different factors. For both outpatient and inpatient visits, the most important factor contributing to a pro-rich distribution was living standard.

Zhang reported that the HIs for the probability of outpatient and inpatient visits among the mid-aged and elderly in 2011 were 0.0373 and 0.1633, respectively [26]. A comparison with our findings implies that the inequity in health care utilization among the mid-aged and elderly had increased from 2011 to 2013. As Li showed, the HIs for the probability of outpatient care and inpatient care in the whole population in 2013 were 0.009 and 0.053, respectively [16]. Compared to the whole population, the distribution of health care utilization among the mid-aged and elder was more pro-rich.

The great share of total inequity, stemming from inequality in probability of health care utilization, implied that the total inequity may be more patient-initiated than doctor-driven. The HI for the total number of outpatient visits was just a little higher than that for the probability of an outpatient visit; while the HI for total number of inpatients visits was a little lower than that for the probability of an inpatient visit. A potential explanation for this finding is that the deductible for inpatient visits was cancelled when patients made a second inpatient visit in one year. Thus, the low income group was less likely to have inpatient visits, but once they had the initial inpatient visit, they seemed to have gone more times in the year. The reimbursement rate and ceiling for outpatient care was low; so the patients had to pay a high out-of-pocket for subsequent outpatient care.

As Lu and Doorslaer found, income skewed the distribution of health care utilization toward the rich [33,34]. This implied that the living standard still greatly impacted the access to health care among the elderly in China. Low income and the high cost of treatment may create financial barriers to making use of health care for older people. Furthermore, with ageing increasing, there will be more and more retired and unemployed people. These people may not have adequate sources of income, and their living standard will probably continue to deteriorate. This may increase the existing inequity in health care utilization.

Although most of the older people in China were covered by health insurance plans, there were great differences in the depth and height of the coverage. Therefore, the effect of health insurance on reducing inequity in health care utilization among the middle-aged and elderly is still limited. The contribution of health insurance varied between plans. The contribution of the URBMI was negative; whereas that of the NRCMS was positive. This implied that the NRCMS did not eliminate the unequal distribution of health care utilization. Wang, Yang, and Zhou also concluded that the URBMI increased the pro-poor inequality in health care utilization and that the effects of NRCMS on decreasing inequality in healthcare utilization were limited [18,25,35,36]. Other studies in Asia have found a pro-rich contribution of health insurance [10,23,34]. These findings may be attributed to a coverage gap between the different health insurance plans, including differences in the benefit packages and co-payments. Zhang suggested that there was a gap in medical utilization between subgroups with different benefit packages and contributions connected with the Chinese multiple health insurance plans [37]. Devaux and Lu also emphasized that high out-of-pocket payments can produce pro-rich inequity [34,38]. Because of a narrow benefit package, the respondents covered by the NRCMS had less access to health care and less frequent visits. The reimbursement rates of the NRCMS were lower, especially for outpatient visits. The respondents had to pay the majority of the outpatient care expenses and half of the inpatient expenditures. The narrow benefit packages and high co-payments of the NRCMS may lead to its high pro-rich contribution, in contrast with the pre-poor contribution of the URBMI. Low-income individuals may be more sensitive to the cost-sharing aspects of health insurance plans. Kim suggested that extending National Health Insurance benefits coverage led to an increase in the utilization of health services across all income groups, but with the greatest increases for the low income group. Thus, extending the benefit coverage reduced the gap in access to health care across socioeconomic groups and improved the income-related equality in health care utilization in South Korea [39]. To decrease inequality, it is necessary to improve the coverage of health care benefits and provide financial protection.

Moreover, chronic disease made a positive contribution to the pro-rich inequity in health care utilization. More than seventy percent of the middle-aged and elderly had a chronic disease and the prevalence rose in recent years. Individuals with a chronic disease are presumed to have greater health care needs. They sought outpatient visits frequently and had heavy economic burdens. However, the benefit packages of the health insurance plans were inpatient-oriented and covered only a limited amount of the expenses of outpatient visits and medication. The medical expenses associated with chronic diseases were paid primarily by the users. Lack of financial protection resulted in barriers to utilizing health care for individuals with chronic diseases and a pro-rich contribution to inequity.

The current study has several strengths. First, using a nationally representative sample of middle-aged and older adults, we examined inequality and horizontal inequity in health care utilization in 2013. This provided the opportunity to examine changes in the inequality in health care utilization. Second, compared to previous studies, this study not only analyzed the inequality in probability but also investigated the inequality in frequency. These findings indicated that the patterns of horizontal inequity in both outpatient and inpatient care utilization were patient-initiated rather than doctor-driven. There were several barriers for the patients in making use of health care, including financial unaffordability and a limited health insurance benefit package.

Finally, this study had several limitations. First, all the information about health care utilization and living standard was self-reported, which may have led to report bias. Second, the failure to distinguish the retired from those not working and missing observations may have led to estimation bias. Last, the study used a cross-sectional analysis, which may lead to common method bias and variance. Thus, caution is required in inferring casual relations. More data sources and methods should be found to control for these biases in further research. Despite these limitations, this study has important policy implications for China towards reducing socioeconomic disparities in health care utilization among middle-aged and older people.

## 5. Conclusions

In conclusion, we confirmed the presence of pro-rich horizontal inequities in both the probability and frequency of health services among middle-aged and older adults in China in the context of universal health insurance coverage. The findings also showed that living standard is the most important factor leading to unequal health care utilization, and the effect of health insurance on reducing the inequality in health care utilization is limited. Therefore, to enhance the equity in health care utilization for the elderly, the government will need to take measures to narrow the gap in living standard and to provide financial support to the low-income group. Moreover, the different health insurance plans should be integrated and the co-payment reduced for older adults. More equitable and effective benefit packages, such as expanding coverage of high-priority services to everyone and outpatient care to chronic disease patients, should also be designed.

## Figures and Tables

**Table 1:** Characteristics of the study participants. UEBMI = Urban Employee Basic Medical Insurance; NRCMS = New Rural Cooperative Medical Scheme; URBMI = Urban Residents Basic Medical Insurance.

Variables	Category	All (n = 13,302)
Dependent variables	At least one outpatient visit in the last month, n (%)	2826 (21.24)
	Total number of outpatient visits, mean (SD)	0.49 ± 1.57
	At least one inpatient visit in the last year, n (%)	1654 (12.43)
	Total number of inpatient visits, mean (SD)	0.18 ± 0.64
Need variables		
Gender	Female ^1^, n (%)	6814 (51.23)
	Male, n (%)	6488 (48.77)
Age	45−59.1, n (%)	7019 (52.77)
	60−74, n (%)	5285 (39.73)
	75+, n (%)	998 (7.50)
Self-reported health status	Very good ^1^, n (%)	651 (4.89)
	Good, n (%)	1547 (11.63)
	Fair, n (%)	4382 (32.94)
	Poor, n (%)	4790 (36.01)
	Very poor, n (%)	1932 (14.52)
Chronic disease	Yes, n (%)	9642 (72.49)
	No ^1^, n (%)	3660 (27.51)
Disability	Yes, n (%)	3050 (22.93)
	No ^1^, n (%)	10,252 (77.07)
Non-need variables		
Health insurance schemes	UEBMI ^1^, n (%)	1416 (10.65)
	URBMI, n (%)	860 (6.47)
	NRCMS, n (%)	9100 (68.41)
	Other health insurance, n (%)	450 (3.38)
	No health insurance, n (%)	424 (3.19)
	Two kinds of health insurance, n (%)	1052 (7.91)
Education level	Illiterate ^1^, n (%)	3187 (23.96)
	Primary school, n (%)	5406 (40.64)
	Middle school, n (%)	2929 (22.02)
	High school, n (%)	1471 (11.06)
	College and above, n (%)	309 (2.32)
Region	East ^1^, n (%)	4575 (34.39)
	Central, n (%)	4391 (33.01)
	West, n (%)	4336 (32.60)
Marital status	Married ^1^, n (%)	11,854 (89.11)
	Divorced, n (%)	149 (1.12)
	Unmarried, n (%)	1299 (9.77)
Occupation status	Agricultural work ^1^, n (%)	6104 (45.89)
	Employed, n (%)	2055 (15.45)
	Self-employed, n (%)	963 (7.24)
	Not working, n (%)	4180 (31.42)
Area	Urban, n (%)	5140 (38.64)
	Rural ^1^, n (%)	8162 (61.36)
Living standards	Pce, mean (SD)	11,177.52 ± 18,359.71

Note: ^1^ Reference group.

**Table 2:** Quintile distributions, inequality, and inequity in health care visits. CI = concentration index; HI = horizontal inequity.

Quintiles	Probability of Outpatient Visits	Total Number of Outpatient Visits	Probability of Inpatient Visits	Total Number of Inpatient Visits
Poorest	0.1819	0.4055	0.0888	0.1261
2	0.2119	0.4386	0.1023	0.1379
3	0.2173	0.5323	0.1246	0.1888
4	0.2201	0.5574	0.1511	0.2202
Richest	0.2650	0.6372	0.1725	0.2483
CI	0.0642 *	0.0780 *	0.1418 *	0.1471 *
HI	0.0714 *	0.0882 *	0.1636 *	0.1596 *

Note: * *p* < 0.05.

**Table 3:** Contribution to inequalities in utilization of outpatient visits.

Variable	Probability			Total Number		
	Coeff ^1^	Cont ^2^	Percent	Coeff ^1^	Cont ^2^	Percent
Need variables		−0.0073	−11.37		−0.0102	−13.08
Male	−0.1143 *	−0.0009	−1.46	−0.3151 **	−0.0016	−2.04
60−74	−0.0652	0.0016	2.55	0.0412	−0.0006	−0.82
75+	−0.1274	0.0014	2.13	−0.0343	0.0002	0.31
Health good	0.3307 **	0.0013	2.09	0.2196	0.0005	0.64
Health fair	0.5247 **	0.0044	6.85	0.7493 **	0.0036	4.67
Health poor	0.7359 **	−0.0008	−1.17	1.2220 **	−0.0007	−0.92
Health very poor	0.9655 **	−0.0165	−25.71	1.5888 **	−0.0136	−17.50
Chronic disease	0.4883 **	0.0033	5.16	0.8124 **	0.0037	4.79
Disability	0.0483	−0.0011	−1.71	0.1226	−0.0017	−2.18
Non-need variables		0.0103	16.04		0.0095	12.18
URBMI	−0.1762	−0.0028	−4.29	−0.3123	−0.0032	−4.12
NRCMS	−0.1221	0.0126	19.71	−0.1020	0.0064	8.26
Other health insurance	−0.1191	−0.0022	−3.37	−0.3513	−0.0041	−5.29
No health insurance	−0.2597 *	0.0005	0.81	−0.0700	0.0001	0.12
Two kinds of health insurance	−0.0772	−0.0009	−1.44	−0.0068	−0.0001	−0.07
Primary school	0.0194	−0.0006	−0.97	0.0307	−0.0006	−0.78
Middle school	0.0189	0.0005	0.77	0.0792	0.0013	1.63
High school	0.0218	0.0009	1.39	0.1361	0.0034	4.36
College and above	−0.0184	−0.0004	−0.57	0.1967	0.0024	3.10
Central	−0.1047 *	0.0015	2.32	−0.1583	0.0014	1.81
West	−0.0083	0.0001	0.17	0.0180	−0.0001	−0.18
Divorced	0.3568	0.0020	3.10	0.7201 *	0.0022	2.79
Unmarried	0.1476	−0.0017	−2.62	0.2010 *	−0.0013	−1.73
Employed	−0.1204	−0.0023	−3.62	−0.2008	−0.0025	−3.17
Self-employed	−0.0787	−0.0009	−1.35	0.0608	0.0004	0.55
Not working	0.0398	0.0020	3.15	0.0400	0.0012	1.60
Urban	0.0205	0.0020	3.11	0.0428	0.0026	3.30
Living standards						
Ln pce ^3^	0.0846 **	0.0587	91.39	0.1313 **	0.0562	72.04

Note: ^1^ Coefficient (coeff); ^2^ Contribution (cont); ^3^ logarithm value of the per capita household expenditure (Ln pce); * *p* < 0.05; ** *p* < 0.01.

**Table 4:** Contribution to the inequalities in utilization of inpatient visits.

Variable	Probability			Total Number		
	Coeff ^1^	Cont ^2^	Percent	Coeff ^1^	Cont ^2^	Percent
Need variables		−0.0217	−15.30		−0.0124	−8.43
Male	0.0999	0.0009	0.64	0.1860 *	0.0008	0.56
60−74	0.0933 *	0.0004	0.28	0.1028 *	0.0002	0.14
75+	0.1065	−0.0030	−2.14	0.1695 *	−0.0023	−1.56
Health good	0.1628	−0.0022	−1.56	0.3166	−0.0019	−1.30
Health fair	0.2360 *	0.0022	1.53	0.4290 *	0.0018	1.23
Health poor	0.5043 **	−0.0006	−0.41	0.8393 **	−0.0004	−0.29
Health very poor	0.8589 **	−0.0187	−13.21	1.4619 **	−0.0109	−7.44
Chronic disease	0.3160 **	0.0024	1.70	0.7588 **	0.0030	2.07
Disability	0.1179 **	−0.0031	−2.17	0.2256 **	−0.0027	−1.85
Non-need variables		0.0058	4.09		−0.0077	−5.24
URBMI	−0.0946	−0.0017	−1.18	−0.1318	−0.0012	−0.80
NRCMS	−0.0038	0.0004	0.31	−0.0396	0.0022	1.48
Other health insurance	−0.2430 *	−0.0045	−3.15	−0.4807 *	−0.0049	−3.34
No health insurance	0.0907	−0.0002	−0.17	0.0503	−0.0001	−0.04
Two kinds of health insurance	0.0605	0.0009	0.60	0.1157	0.0008	0.52
Primary school	0.0678	−0.0024	−1.72	0.0638	−0.0011	−0.75
Middle school	0.0625	0.0018	1.30	−0.0370	−0.0005	−0.35
High school	−0.0268	−0.0012	−0.84	−0.2242	−0.0049	−3.32
College and above	−0.0018	0	−0.03	−0.1769	−0.0019	−1.29
Central	0.0305	−0.0005	−0.35	0.2350 **	−0.0018	−1.24
West	0.1572 **	−0.0023	−1.65	0.4117 **	−0.0028	−1.93
Divorced	−0.0220	−0.0001	−0.08	−0.0166	0	−0.03
Unmarried	0.0385	−0.0005	−0.33	0.0626	−0.0004	−0.25
Employed	−0.2095 **	−0.0043	−3.01	−0.3614 **	−0.0039	−2.63
Self-employed	0.0636	0.0008	0.59	0.1327	0.0008	0.55
Not working	0.2576 **	0.0152	10.69	0.5015 **	0.0136	9.26
Urban	0.0403	0.0044	3.09	−0.0310	−0.0016	−1.10
Living standards						
Ln pce ^3^	0.1717 **	0.1325	93.45	0.3046 **	0.1136	77.19

Note: ^1^ Coefficient (coeff); ^2^ Contribution (cont); ^3^ logarithm value of the per capita household expenditure (Ln pce); * *p* < 0.05; ** *p* < 0.01.

## References

[1] Penning M.J., Zheng C. (2016). Income inequities in health care utilization among adults aged 50 and older. Can. J. Aging.

[2] Zhang X., Wu Q., Shao Y., Fu W., Liu G., Coyte P.C. (2015). Socioeconomic inequities in health care utilization in China. Asia Pac. J. Public Health.

[3] Braveman P. (2006). Health disparities and health equity: Concepts and measurement. Annu. Rev Public Health.

[4] Tang S., Meng Q., Chen L., Bekedam H., Evans T., Whitehead M. (2008). Tackling the challenges to health equity in China. Lancet.

[5] Van Doorslaer E., Koolman X., Jones A.M. (2004). Explaining income-related inequalities in doctor utilisation in Europe. Health Econ..

[6] Macinko J., Lima-Costa M.F. (2012). Horizontal equity in health care utilization in Brazil, 1998–2008. Int. J. Equity Health.

[7] Vikum E., Krokstad S., Westin S. (2012). Socioeconomic inequalities in health care utilisation in Norway: The population-based HUNT3 survey. Int. J. Equity Health.

[8] Barraza-Llorens M., Panopoulou G., Diaz B.Y. (2013). Income-related inequalities and inequities in health and health care utilization in Mexico, 2000–2006. Rev. Panam Salud Publica.

[9] Nunez A., Chi C. (2013). Equity in health care utilization in Chile. Int. J. Equity Health.

[10] Duy Kien V., Van Minh H., Bao Giang K., Weinehall L., Ng N. (2014). Horizontal inequity in public health care service utilization for non-communicable diseases in urban Vietnam. Glob. Health Action.

[11] Devaux M. (2015). Income-related inequalities and inequities in health care services utilisation in 18 selected OECD countries. Eur. J. Health Econ..

[12] Dorjdagva J., Batbaatar E., Dorjsuren B., Kauhanen J. (2015). Income-related inequalities in health care utilization in Mongolia, 2007/2008–2012. Int. J. Equity Health.

[13] Park J.M. (2016). Equity in the utilization of physician and inpatient hospital services: Evidence from Korean health panel survey. Int. J. Equity Health.

[14] Flato H., Zhang H. (2016). Inequity in level of healthcare utilization before and after universal health coverage reforms in China: Evidence from household surveys in Sichuan province. Int. J. Equity Health.

[15] Liu M., Zhang Q., Lu M., Kwon C.S., Quan H. (2007). Rural and urban disparity in health services utilization in China. Med. Care.

[16] Li Y. (2016). Research of Prediction and Equity of Utilization of Health Services of Residents in China. Master’s Thesis.

[17] Zhou Z., Gao J., Fox A., Rao K., Xu K., Xu L., Zhang Y. (2011). Measuring the equity of inpatient utilization in Chinese rural areas. BMC Health Serv. Res..

[18] Zhou Z., Su Y., Gao J., Campbell B., Zhu Z., Xu L., Zhang Y. (2013). Assessing equity of healthcare utilization in rural China: Results from nationally representative surveys from 1993 to 2008. Int. J. Equity Health.

[19] World Health Organization (WHO) (2015). World Report on Ageing and Health.

[20] Rechel B., Grundy E., Robine J.M., Cylus J., Mackenbach J.P., Knai C., McKee M. (2013). Ageing in the European Union. Lancet.

[21] The Office of China National Committee on Aging (2015). The general research report of Chinese strategic for dealing with population aging. Sci. Res. Aging.

[22] Crespo-Cebada E., Urbanos-Garrido R.M. (2012). Equity and equality in the use of GP services for elderly people: The Spanish case. Health Policy.

[23] Joe W., Rudra S., Subramanian S.V. (2015). Horizontal inequity in elderly health care utilization: Evidence from India. J. Korean Med. Sci..

[24] Terraneo M. (2015). Inequities in health care utilization by people aged 50+: Evidence from 12 European countries. Soc. Sci. Med..

[25] Wang Y., Wang J., Maitland E., Zhao Y.H., Nicholas S., Lu M.S. (2012). Growing old before growing rich: Inequality in health service utilization among the mid-aged and elderly in Gansu and Zhejiang provinces, China. BMC Health Serv. Res..

[26] Zhang G. (2013). Explaining Economic Status Related Inequity of Health Care Utilization among the Mid-Aged and Elderly in China. Master’s Thesis.

[27] The China Health and Retirement Longitudinal Study (CHARLS). http://charls.pku.edu.cn/zh-CN.

[28] Zhao Y., Hu Y., Smith J.P., Strauss J., Yang G. (2014). Cohort profile: The China health and retirement longitudinal study (CHARLS). Int. J. Epidemiol..

[29] O’Donnell O., Doorslaer E.V., Wagstaff A., Lindelow M. (2008). Analyzing Health Equit Using Household Survey Data: A Guide to Techniques and Their Implementation.

[30] Wagstaff A., Paci P., van Doorslaer E. (1991). On the measurement of inequalities in health. Soc. Sci. Med..

[31] Wagstaff A., van Doorslaer E. (2000). Measuring and testing for inequity in the delivery of health care. J. Hum. Resour..

[32] Wagstaff A., van Doorslaer E., Watanabe N. (2003). On decomposing the causes of health sector inequalities with an application to malnutrition inequalities in Vietnam. J. Econ..

[33] Van Doorslaer E., Masseria C. (2004). Income-Related Inequality in the Use of Medical Care in 21 OECD Countries.

[34] Lu J.F., Leung G.M., Kwon S., Tin K.Y., Van Doorslaer E., O’Donnell O. (2007). Horizontal equity in health care utilization evidence from three high-income Asian economies. Soc. Sci. Med..

[35] Yang W. (2013). China’s new cooperative medical scheme and equity in access to health care: Evidence from a longitudinal household survey. Int. J. Equity Health.

[36] Zhou Z., Zhu L., Zhou Z., Li Z., Gao J., Chen G. (2014). The effects of China’s urban basic medical insurance schemes on the equity of health service utilisation: Evidence from Shaanxi province. Int. J. Equity Health.

[37] Zhang C.Y., Hashimoto H. (2015). How do patients and providers react to different incentives in the Chinese multiple health security systems?. Chin. Med. J..

[38] Devaux M., De Looper M. (2012). Income-Related Inequalities in Health Service Utilisation in 19 OECD Countries.

[39] Kim S., Kwon S. (2014). The effect of extension of benefit coverage for cancer patients on health care utilization across different income groups in South Korea. Int. J. Health Care Financ. Econ..

